# Selections and Abstracts

**Published:** 1903-04

**Authors:** 


### SELECTIONS AND ABSTRACTS.

### Pathology of Pulmonary Tuberculosis.

In order for infection to take place the tubercle bacilli must gain access to the intervesicular connective tissue, forming tubercles by proliferation of fixed connective tissue cells. The further spread of the bacilli is mainly by way of the lymphatics; sometimes in phagocytes and rarely by the blood, with general dissemination of the tubercular poison. There are frequently initial ulcers on the bronchial mucous membrane. In children particularly, pulmonary tuberculosis is often secondary to tuberculosis of the bronchial glands resulting from infection through the tonsils, and the earliest lesion is frequently found in the neighborhood of the hilum. Generally speaking, tubercle formation begins rather deeply and extends toward the periphery. When infection takes place primarily through the intestinal tract, the mesenteric glands are commonly first involved. It is reasonable to believe that autoinfection may take place by the same route.

The chief factors in combating the lodgment and multiplication of bacteria in the body are the ciliated epithelial lining of the upper respiratory tract, the bactericidal action of the nasal mucoso and the gastric juice, and the phagocytic and antitoxic properties of the blood.

The pathologic unit of tuberculosis is the submiliary tubercles, minute nodules about 1–60 inch in diameter. Miliary tubercles are made up of from ten to fifty of the smaller growths, and appear to the naked eye as gray, translucent bodies, the size of a pinhead or a hempseed, with a white center. They are nearly non-vascular, becoming dry, opaque, white or yellow caseous bodies. The presence of tubercles in the tissues may produce caseation, ulceration, sclerosis or calcification—one or more, or all combined.

Tubercles are made up chiefly of a reticulum of cuboidal epithelioid cells with vesiculated nuclei. These cells are derived from parenchyma cells, from the endothelia of blood and lymph vessels, and the epithelia of serous membranes. Wandering leucocytes invade the connective tissue matrix, and constitute the lymphoid cells of the tubercles.

Large, composite "giant cells" are characteristic, though not pathognomonic, of tuberculosis. They may develop from epithelia or endothelia by incomplete segmentation, owing to deficient nutrition, the nuclei, but not the protoplasm, dividing. Their number is in inverse relation to the tubercle bacilli, which are commonly arranged within and around the margin of the "giant cell."

Dissolution of tubercles is nearly always due to secondary infection by pyogenic germs. Under favorable circumstances, the vascular zone surrounding the tubercle undergoes fibrous change, leading to cicatrization or encapsulation of the caseous, or better still, the calcified nodule. On the other hand, when the agminated masses of round cells and bacilli are large, they are liable by confluence, through cutting off nutrition, to undergo coagulation necrosis and eventually caseation, forming the so-called tubercular abscesses, which, on evacuation through a bronchus, leave cavities. These are lined with a layer of necrosed caseous tubercular material, bounded externally by a wall of granulation tissue and lymphoid elements. Cavities are sometimes traversed by more or less corroded blood-vessels or bronchioles.

Quiescent, or "healed in" tubercles are those in which the tubercular process has been self-limited by the development of a dense fibrous boundary wall, in which is retained the more solid caseous detritus or calcareous mass, still including as a rule potential bacilli, which may break forth again with virulence under favoring conditions.

The so-called pretubercular stage corresponds with the period of eruption of tubercles before the appearance in the sputa of tubercle bacilli, which may be absent for months in the incipient, or even an advanced stage of the disease. They can be present in the expectoration only when there is free communication between the softened tubercular focus and the bronchial apparatus. A few bacilli alternating with large numbers may be due to the occasional opening and evacuation of large cavities. The clumping of granular bacilli indicates a tendency to breaking down of tissues and formation of cavities.

Tubercular deposits take place first as a rule and most abundantly at the tip of the lung, along its borders and posterior aspect. When infection is direct, by the air passages, a bronchopneumonic and lobular tuberculosis is set up, followed by caseation of inflammatory products, and terminating either in sclerosis with limitation of morbid products, or in dissemination by lymph and blood when the connective tissue is exposed by breaking down of the intervening wall.

The extension of the tubercular process may be slow or rapid, steady or intermittent. The disease is often widely disseminated before any subjective symptoms are noted. When it has extended so as to involve a whole lobe, the symptoms often ameliorate, and the so-called latent stage of arrested tuberculosis supervenes. In favorable instances a case of advanced phthisis may also enter on a quiescent stage after the discharge, absorption or inspiration of the abscess contents, or when formed cavities become clean and dry. Usually, however, the tendency is to successive eruptions of tubercles during or soon succeeding excavation.

Phthisis, in a strictly pathological sense, does not begin until the caseous tubercles commence to soften and break down. Acute diseases occurring during a latent tuberculosis are frequently followed by phthisis, since they check the natural encapsulating process of cure and set up liquefactive changes.

Toward the fatal end of phthisis the pathological changes may include a variety of lesions, such as miliary tuberculosis; caseous, fibroid and calcareous degeneration; pneumonic infiltration; bronchial catarrh and ulceration; areas softening into excavations; and open and closed cavities in all stages of inflammation, ulceration and cicatrization.

The common associated changes of chronic ulcerative phthisis are catarrhal pneumonia, bronchopneumonia, purulent bronchial catarrh and pleuritis, and proliferation of the interstitial connective tissue. Pneumonias from mixed infection are markedly severe, causing a rapid advance of the tubercular disease and early decline. Tubercular children are particularly liable to attacks of localized pneumonia around the tubercular area.

The acute miliary, pneumonic and bronchopneumonic types, the ordinary caseous tuberculosis (chronic ulcerative phthisis), and the chronic fibroid form of the disease, represent, generally speaking, different degrees of infection and of individual resistance and reaction.—*Denver Medical Times.*

---

### DISEASE IN FICTION.

There have been many more or less correct communications as to the employment of disease in fiction. Conan Doyle has made demonstrably erroneous statements in this particular, despite his medical training. The errors are the more singular because they can be corrected from Doyle's English contemporaries in fiction.

The *Medical Document* demonstrates a curious ignorance of the necessity for impressionist views of disease in fiction. "A curious paper," remarks one of Doyle's physicians, "might be read at a medical meeting about the uses of medicine in fiction, relating to what folk die of and what diseases are made most use of in novels. Some are worn to pieces, and others, which are equally common in real life, are never mentioned. Typhoid is fairly frequent, but scarlet fever is unknown. Heart disease is common, but then heart disease as we know it is usually the sequel of some foregoing disease, of which we never hear anything in the romance. Then there is the mysterious malady called brain fever, which always attacks the heroine after a crisis, but which is unknown under the name to the text-books. People, when they are overexcited in novels, fall down in a fit. In a fairly large experience I have never known any one to do so in real life. The small complaints simply don't exist. Nobody ever gets shingles or quinsy or mumps in a novel. All the diseases, too, belong to the upper part of the body. The novelist never strikes below the belt."

It is singular that Doyle, a physician novelist, should fail to recognize the necessity in fiction of portraying disease and morbid states through the impressions and in the language of laymen. The artist, in accordance with the literary canon, must present facts as seen by minds of the character depicted, in language of the station in life of the character. For this reason clinical accuracy in diction and in description would be manifestly absurd and pedantic. Heart disease and disorder, for example, are frequent outcomes of unsuspected conditions; to describe them otherwise in fiction would be inaccurate and pedantically absurd. Furthermore, cardiac disorder frequently results from nervous and mental strain, conditions which are but too frequently ignored by physicians.

The "mysterious malady called brain fever" has been frequently recognized in medicine as "nervous typhod," confusional insanity, typhomania, meningism, and allied states, where there is a confusional quasi-delirious mental disturbance. Doyle's physician does not recognize this because, like most medical philistines, he labels it meningitis and regards it as a furibund state always with demonstrable pathologic lesions. The always demonstrable pathologic lesion notion has long been a detriment to medicine and therapeusis, leading to errors in prognosis and treatment. The biochemic view urged by alienists during the eighties is now becoming dominant.

The curious error as to scarlet fever indicates Doyle's shallow dilettante impressionism. Not to speak of other very accessible instances, Wilkie Collins employs scarlet fever as part of the plot in "Blind Love." The statement that people do not faint and display convulsive phenomena after overexcitement is demonstrably erroneous. Such conditions occur where autotoxemia is previously present. Autotoxemia is hardly a describable state through the mentality of the ordinary character of fiction. Epilepsy of the true type plays no little part in the evolution of various mental states leading to crime: Shakespeare (whose chief publisher issued more than one work on the brain and nerves) seems to have observed post-epileptic insanity and put upon it the popular interpretation, for he remarks in "Othello" (Act IV., Scene I.):

Iago: "My lord is fallen into an epilepsy;
This is his second fit; he had one yesterday."
Cassio: "Rub him about the temples."
Iago: "No, forbear;
The lethargy must have its quiet course,
If not, he foams at mouth, and by and by
Breaks out to savage madness."

Dickens makes Monks, the villain of "Oliver Twist," an epileptic. Epilepsy and its silver nitrate therapeusis is an essential part of the plot of Wilkie Collins's "Poor Miss Finch." The hero of the story (Oscar Dubourg) is assaulted by burglars and becomes in consequence an epileptic. He is enamored of Miss Finch, who in consequence of cataract has become blind at an early age. She has an intense fear of darkness and dark people. Collins adopts the theory of the early nineteenth century that silver nitrate pushed to saturation is a cure for epilepsy. This view persisted quite late in the seventies, as witness the many "blue" epileptics to be found in urban county insane hospitals. Knowing the full consequences, Oscar Dubourg has himself saturated with silver nitrate to the extent of producing the usual bluish-black complexion. Miss Finch hears somebody call Oscar the "blue man." She is told this refers not to Oscar, but to his twin brother, who also falls in love with her. A German oculist, who completely disregards asepsis, operates for cataract and secures sight for Miss Finch. In her desire to see Oscar she tears off the bandage and embraces Oscar's brother, disregarding Oscar as the "blue man." The entanglement thus created is removed by Miss Finch losing her prejudice against darkness, and finally losing her sight from overexcitement. She becomes blind, but Oscar remains cured of his epilepsy.

Leprosy plays a part in "Lady Jezebel," by Fergus Hume. Here the popular notion as to the very infectious nature of leprosy, much cultivated by medical sensationalists, is adopted. The leading character in the story plays the part of an adventuress in India in the days before the Mutiny, and secures the diamond heirlooms of an indian rajah. His son, attempting to reclaim the diamonds, visits England and becomes a guest of the husband of the adventuress. The husband and the young rajah become addicted to cannabis indica. The rajah infects the Englishman with leprosy; after an orgy the Englishman drives the Indian into a swamp, where he perishes. The Englishman becomes insane, and is shut up in a mansion by his wife, who remarries. He escapes from his keepers, and finding the second husband beating the wife, kills him. To avoid publicity, the murder is laid on an innocent man. The story winds up with the leper firing the house and carrying off his wife to perish with him in a marine swamp.

Epilepsy plays a part in European fiction as well as fiction of the English-speaking nations. George Sand's Consuelo is founded in part on an actual case of epilepsy, where delusions of memory persisting from epileptic mental states were accepted as memories of previous existence. The same employment of epilepsy occurs in Maurice Sand's Callirhoé. Of the developments of Morel's degeneracy views in Zola's Rougon-Macquart series it is unnecessary to speak at length.—*Medicine.*

---

### LOBAR PNEUMONIA.

Notwithstanding the enormous amount of literature contributed to medical journals upon the subject of pneumonia, the understanding of this disease, especially its effective treatment, has not been materially increased. Like rheumatism the term pneumonia is by no means a definite name of a certain pathologic condition, but covers a variety of bacterial infections of the lungs, and this perhaps accounts for its verbose but well-nigh profitless discussion.

In the February number of *Medicine*, J. M. Anders endeavors to give some practical thoughts on pneumonia. He quotes the census statistics as showing an increase of the mortality from 186 to 191 per 100,000 of population in the last decade. This he accounts for by the epidemic prevalence of influenza, overcrowding in cities, sedentary habits and the enormous consumption of malt liquors impairing renal function.

Pneumonia due to the streptococcus has become common since the advent of influenza. Its symptomatology and course differ from that caused by the pneumococcus. This is also true of pneumonitis caused by the bacillus coli and various other organisms, and Anders quotes Aufrecht approvingly as saying that the "exact limitation of the various forms will be possible only when the classification is carried out according to the bacterial causes." He thinks that toxemia is an element that has received too little attention in the past. The fact that resolution in this disease is probably occasioned by a ferment is a recent contribution to the pathology of the affection.

Under symptomatology he dwells upon the aberrant forms. In pure streptococcus pneumonia associated with influenza we have septic manifestations and the looked-for irregular fever and sweats, with profound prostration. "The patient is desperately ill of his pneumonia for days before the physical signs, which are those of bronchopneumonia, put in an appearance; moreover, these are quite indefinite in some cases throughout their entire course, and in others only until the disease is fully developed. A serpiginous tendency is often observed, and instead of a crisis on the seventh or ninth days the condition is apt to be protracted, and the fever to terminate by lysis. This is true of those cases that recover; sadly euough, many succumb early, not so much to the effect of the local inflammatory lesion as the general infection—the toxemia. On the other hand, cases of pneumococcus infection, secondary to influenza, may also run a protracted course, without crisis."

Anders has met with cases of pneumonia that answered clinically to that variety known as typhoid pneumonia. Examination of the sputum showed many of them to be due to the pneumococcus. The asthenic pneumonia of Leichtenstern has also become more prevalent recently.

In the treatment of pneumonia, Anders thinks that the best means to prevent the increasing asthenia which is prone to develop as the crisis is approached are nutritious diet, free use of alcoholic stimulants, digitalis, strychnin and sulphuric ether administered hypodermically. He has much confidence in oxygen by inhalation, and normal saline solution subcutaneously. Cold water compresses, as recommended by Simon Baruch, reduce temperature, stimulate the respiration and circulation, and relieve pain. Venesection may be done upon robust patients. For pulmonary edema, dry cupping—one to two dozen or more—is excellent. Nothing is said of serum treatment, salicylates or creosote.—*St. Louis Medical Review.*

